# Marine Bacteria Display Different Escape Mechanisms When Facing Their Protozoan Predators

**DOI:** 10.3390/microorganisms8121982

**Published:** 2020-12-12

**Authors:** Richard Guillonneau, Claudine Baraquet, Maëlle Molmeret

**Affiliations:** 1 Laboratoire MAPIEM, EA4323, Université de Toulon, 83130 La Garde, France; richard.guillonneau@warwick.ac.uk (R.G.); baraquet@univ-tln.fr (C.B.); 2 School of Life Sciences, University of Warwick, Coventry CV4 7AL, UK

**Keywords:** marine bacteria, amoeba, intracellular trafficking, biofilm, escape mechanism, survival

## Abstract

Free-living amoeba are members of microbial communities such as biofilms in terrestrial, fresh, and marine habitats. Although they are known to live in close association with bacteria in many ecosystems such as biofilms, they are considered to be major bacterial predators in many ecosystems. Little is known on the relationship between protozoa and marine bacteria in microbial communities, more precisely on how bacteria are able survive in environmental niches where these bacterial grazers also live. The objective of this work is to study the interaction between the axenized ubiquitous amoeba *Acanthamoeba castellanii* and four marine bacteria isolated from immersed biofilm, in order to evaluate if they would be all grazed upon by amoeba or if they would be able to survive in the presence of their predator. At a low bacteria-to-amoeba ratio, we show that each bacterium is phagocytized and follows a singular intracellular path within this host cell, which appears to delay or to prevent bacterial digestion. In particular, one of the bacteria was found in the amoeba nucleolar compartment whereas another strain was expelled from the amoeba in vesicles. We then looked at the fate of the bacteria grown in a higher bacteria-to-amoeba ratio, as a preformed mono- or multi-species biofilm in the presence of *A. castellanii*. We show that all biofilms were subjected to detachment from the surface in the presence of the amoeba or its supernatant. Overall, these results show that bacteria, when facing the same predator, exhibit a variety of escape mechanisms at the cellular and population level, when we could have expected a simple bacterial grazing. Therefore, this study unravels new insights into the survival of environmental bacteria when facing predators that they could encounter in the same microbial communities.

## 1. Introduction

Protozoa such as free-living amoeba are important members of microbial communities in all terrestrial, fresh, and marine environments. Protozoa are known to live in close association with bacteria in many ecosystems such as biofilms [1]. Predation by protozoa is considered to be the leading cause of bacterial death [2]. In current times, studies are mostly focused on understanding the physiology and regulation of sessile bacteria without sufficiently taking into consideration other major players of microbial communities [1]. The close association of bacteria and protozoa in biofilm and their long co-evolutionary history are thought to give rise to a series of bacterial adaptations ensuring survival and coexistence [3,4,5]. This aspect has been particularly well studied when protozoa interact with pathogenic bacteria, which is best illustrated with the protozoa-*Legionella pneumophila* relationship. Protozoa are indeed considered like a “Trojan horse” for the selection of pathogenic bacteria [6,7]. In marine environments, protozoa could have a major impact on the selection of bacteria in biofilm ecosystem and on the selection of pathogens in aquaculture. Studies on the coexistence of bacteria and protozoa including amoeba in the environment are emerging but little is known on the establishment of this type of relationship in particular when marine microorganisms are considered. Overall, very few studies have focused on answering the following conundrum: If protozoa are bacterial grazers and part of the biofilm microbial communities, how do bacteria survive in the presence of their predators in biofilms?

The first interactions between amoeba and bacteria that have been reported involved human pathogenic bacteria [7,8,9,10,11]. Since then, various potential routes of bacterial adaptations against predation by protozoa have been described. Two types of bacterial adaption or resistance towards amoeba has been proposed: pre-ingestional and post-ingestional with various adaptation strategies associated with bacterial size or grouping, bacterial speed, amoeba surface masking, digestional resistance, toxin release, or intracellular growth [3,5]. In recent years, original interactions have been discovered in marine environments. For instance, several intranuclear bacterial symbionts of marine amoeba have been identified (reviewed in [12]). While different types of interactions have been described with various bacteria, mostly human pathogens, very few studies have been interested in interactions between marine bacteria (mainly non-pathogenic bacteria) with protozoa, their intracellular fate, and the potential outcome within the microbial communities. Except for the few existing amoeba models, very few protozoa have been isolated and axenized from the environment, despite an important diversity of amoeboid protists present in the marine environment [13].

Different marine bacteria have been isolated from biofilms recovered from the bay of Toulon, France [14,15]. These bacteria, *Persicivirga* (*Nonlabens*) *mediterranea* (TC4), *Polaribacter* sp. (TC5), and two strains of *Shewanella* (TC10 and TC11), initially selected on the basis of phenotypical differences [15,16,17], have been recently studied in multispecies biofilms [18]. It was found that competitive and cooperative interactions take place within multispecies biofilms. In this study, we investigated the interactions between these four different marine bacterial strains and an amoeba susceptible to occupy the same ecological niche, the ubiquitous axenized amoeba, *Acanthamoeba castellanii. A. castellanii* has been isolated from various sources including soil, natural, and tap waters as well as marine samples [19,20,21,22] and has been used as a model organism for bacteria–amoeba interaction studies with different bacteria, including marine bacteria [10,23,24,25]. When we looked at the interactions at a low bacteria-to-amoeba ratio, we showed that, first, all the bacteria were phagocytized within the protozoan cells and that bacterial uptake occurred at very different efficiencies; second, none of them were totally eliminated by *A. castellanii* during the 48 h incubation, even when inoculated at low concentration; third and most importantly, each bacteria followed a singular intracellular fate. Among these intracellular paths, *Shewanella* sp. TC11 appeared to be expelled from the amoeba in vesicles at 24 h. We also made the original observation of an intranucleolar bacteria (*P. mediterranea* TC4) within a eukaryotic host cell. When we studied the interaction between preformed monospecies and multispecies biofilms and *A. castellanii*, which correspond to a higher bacteria-to-amoeba ratio, we showed that, first, all biofilms detached from the surface during the 24 h interaction with amoeba; second, the amoeba supernatant triggered the detachment of two of the bacterial biofilms from the surface; and third, *Polaribacter* sp. TC5, which have been shown to display inhibitory effects toward other bacteria in multispecies biofilms [18], triggered a cytotoxicity effect on the amoeba. Therefore, our findings show that protozoan interactions with bacteria considered as non-pathogenic can unravel different intracellular paths and escape mechanisms when we could have expected a quick bacterial grazing for all of them. This study highlights the diversity of interactions between eukaryotic cells and bacteria that could exist in the marine environment and how bacteria survive in the presence of their predator through various escape mechanisms at the cellular or at the population level. 

## 2. Materials and Methods 

### 2.1. Bacterial and Protozoan Strains

Four marine bacterial strains were used in this study: *Persicivirga (Nonlabens) mediterranea* TC4 (Toulon Collection), *Polaribacter* sp. TC5, and two different species of *Shewanella*, *Shewanella* sp. TC10, and *Shewanella* sp. TC11. These strains were isolated from surfaces immerged in the bay of Toulon and belonged to different species as shown by their phylogenetic position [14,15]. All bacterial strains were grown in Väätänen Nine-Salt Solution (VNSS) [26] at 20 °C, with shaking at 120 rpm. When the TC10 strain harbored the pX5 plasmid encoding green fluorescent protein (GFP) (kindly constructed by Dr. Aurore Puymège), the VNSS medium was supplemented with 6 µg/mL of chloramphenicol. 

*A. castellanii* is an axenized environmental isolate that was provided by Pr. F. Pernin (Institut des Sciences Pharmaceutiques et Biologiques-Faculté de Pharmacie-Université Lyon1, Lyon, France). The amoeba, whose axenization implies that they do not require any addition of bacteria for growth, were grown into 175 cm² tissue culture flasks (Nunc™, Thermo Scientific, Roskilde, Denmark) containing 30 mL of PYG (Peptone, Yeast-extract, Glucose) at 20 °C. The coculture assays were performed in the Nine-Salt Solution (NSS) [1,26]. 

### 2.2. Bacterial Labeling

TC10 pX5-GFP was previously constructed by conjugation using *Escherichia coli* WM3064 as a donor strain [18]. Because transformation and conjugation assays of the bacterial strains only resulted in the creation of TC10 pX5-GFP despite numerous different conditions tested on the strains, 3 polyclonal antibodies directed against the 3 other strains, TC4, TC5, and TC11, were produced (ACRIS Antibodies GmbH, Herford, Germany) for labeling purposes [18]. TC4, TC5, and TC11 polyclonal antibodies were respectively designed into chicken, goat, and rabbit hosts. The construction and specificity of antibodies between them as well as optimizations of the parameters were tested and completed in a prior study [18]. The absence of counterstain against *A. castelanii* was performed (Supporting information Figure S1). 

### 2.3. Bacterial and Amoeba Enumeration in NSS

Growth kinetics in NSS of amoeba was performed every 24 h over a time course of 96 h directly on 175 cm^2^ tissue culture flasks using an inverted optical Motic AE21 microscope (Motic, Kowloon, Hong Kong). Evaluation of bacterial concentration was performed at the same time points by measuring absorbance at OD_600nm_.

### 2.4. Bacterial Survival during A. castellanii Co-Incubation

Amoebae suspensions obtained by scrapping culture flasks (Corning Incorporated Costar^®^, New York, NY, USA) were inoculated into 96 transparent well plates at 1.10^5^ cells per well. Plates were incubated for 12 h at 20 °C to initiate amoebae adhesion at the bottom of the wells. Post exponentially grown bacteria suspended in NSS were inoculated on amoebae monolayers at a multiplicity of infection (MOI) of 10 or 100. Low speed centrifugation (5 min at 900× g) was used to slowly move the bacteria down toward the adhered protozoan cells, and to initiate and synchronize physical interaction between bacteria and amoeba as it is usually performed in studies of bacteria–host cell interactions [27,28,29,30,31]. After 3 h of contact time, the samples were washed off 3 times with NSS to remove extracellular bacteria and start the kinetics study at T0h, T24h and T48h. At each time point of the kinetic, host cell samples, treated with Triton^TM^ X-100 (0.05%) for 15 min on ice or mechanically lysed by mixing up and down with syringe and needles (25 g), were submitted to serial dilutions before plating 5 µL drops on VNSS agar plates for the estimation of the intracellular bacteria concentration. 

### 2.5. Cytotoxicity Assay against A. castellanii

Bacteria and amoeba were prepared as previously described except that 24 well plates (Corning Incorporated Costar^®^, New York, NY, USA) containing a sterilized glass coverslip into each well were used and that 5 × 10^5^ cells per well with the corresponding MOI 100 for the bacteria were added in each well. After 24 h, bacteria-associated amoebae were stained with propidium iodide (Thermo Fisher Scientific, Waltham, MA, USA) according to the manufacturer’s instructions followed by 20 min incubation at room temperature in the dark. Samples were then washed three times with NSS before formalin fixation and mounting on a slide with a drop of ProLong™ Diamond Antifade (Molecular Probes™, Eugene, OR, USA). The percentage of live or dead cells was determined using confocal laser scanning microscopy (CLSM) (Zeiss LSM 510). A negative control corresponding to the non-infected amoeba and a positive control corresponding to the non-infected amoeba treated with Formalin 3.7% and Triton^TM^ X-100 (0.05%) to kill the cells were prepared before propidium iodide addition and subsequent fixation and mounting procedures. 

### 2.6. Bacterial Localization within A. castellanii Using CLSM

Bacteria and amoebae were prepared as previously described except that 24 well plates (Corning Incorporated Costar^®^, New York, NY, USA) containing a sterilized glass coverslip into each well were used and 5 × 10^5^ amoeba cells were added in each well. Bacteria were inoculated at both MOI 10 and MOI 100. At T0 h, T24 h, and T48 h, cells were fixed using Formalin 3.7% for 1 h for amoeba-associated TC10 pX5-GFP and TC5 strains, and using acetone/methanol (1:1, v:v) for 20 min at −20 °C for the samples containing amoeba and TC4 or TC11. The permeabilization steps were performed using Triton^TM^ X-100 (0.05%) for 15 min or using Tween^®^ 20 (0.5%) for 20 min in order for the TC4 antibodies to access the nucleus. For the immunostaining, samples were blocked with bovine serum albumin (BSA) 3% (Acros organics, Geel, Belgium) in phosphate buffer saline (PBS) 1X overnight at 4 °C. The primary antibodies were added for 1 h in BSA 3% at 1/300 for chicken-anti-TC4, 1/100 for goat-anti-TC5, and 1/300 for rabbit-anti-TC11. After a second blocking step of 2 h at room temperature, secondary antibodies were added in BSA 3% with 4′,6-diamidino-2-phénylindole (DAPI) at 5µg/mL (Sigma-aldrich, Darmstadt, Germany), at a concentration of 1/4000 for goat anti-chicken IgY (H + L) conjugated to fluorescein isothiocyanate (FITC) (Invitrogen™, Waltham, MA, USA) or to a concentration of 1/500 for goat anti-chicken IgG coupled to Alexa Fluor 405 (Abcam, Cambridge, UK) for 1 h, at a concentration of 1/2000 for donkey anti-goat IgG (H + L) coupled to Alexa Fluor 633 (Invitrogen™, Waltham, MA, USA) for 1 h, at a concentration of 45 µL/mL for donkey anti-rabbit IgG coupled to Alexa Fluor 594 for 30 min, and at a concentration of 1/200 for mouse anti-rabbit IgG coupled to CruzFluor™ 488. Finally, the coverslips were mounted with a drop of ProLong™ Diamond Antifade before observation using CLSM.

### 2.7. Colocalization Studies with Intracellular Organelles

The bacteria and the amoeba were prepared at MOI 100, as described above. The colocalization study of TC4, TC5, TC10, and TC11 strains with acid endosomes was performed at T0 h, T24 h and T48 h using 40 µg/mL of pHrodo™ Red Dextran (Molecular probes™, Eugene, OR, USA) [32]. Samples were incubated for 20 min at 37 °C. The colocalization with the nucleolus was carried out using mouse anti-nucleolin primary antibody (C-23(MS-3): sc-8031) (Santa Cruz biotechnology, Dallas, TX, USA) at 1/50 for 1 h as previously performed [33]. The secondary mouse antibody IgGκ (m-IgGκ BP-CFL 594: sc-516178) (Santa Cruz biotechnology, Dallas, TX, USA) conjugated to CruzFluor™ 594 was applied for 1 h at 1/50.

### 2.8. Bacterial Localization within A. castellanii Using Transmission Electron Microscopy (TEM)

Coculture experiments took place in 24 well plates at MOI 100. At T0 h, T24 h, and T48 h, samples were fixed in 3.5% glutaraldehyde for 45 min at 4 °C in NSS and post-fixed with 1% osmium tetroxide (OsO_4_) in 0.1 M sodium Sorenson’s buffer for 1 h. Dehydration in increasing ethanol concentration solutions was followed by direct embedding of the samples from the surface in LX112 resin (Ted Pella, Redding, CA, USA). Ultrathin sections were stained with uranyl acetate followed or not by lead citrate at the CIQLE (Centre d’Imagerie Quantitative Lyon Est, platform of UCBL Lyon1 University) or at the PiCSL-FBI core facility (IBDM UMR CNRS 7288, Aix-Marseille University) and examined with a Tecnai G2 electron microscope (FEI, Hillsboro, OR, USA) operating at 200 kV at the PiCSL-FBI core facility.

### 2.9. Biofilm Formation

Bacterial biofilms were developed into 96-well microtiter plates (Corning Incorporated Costar^®^, New York, NY, USA) with 200 μL of bacteria harvested in post exponential growth phase in VNSS and suspended in NSS at an OD_600nm_ of 0.4 into each well [18,34]. After 15 h, 24 h, 48 h, and 72 h of development in static conditions and a temperature of 20 °C, samples were washed three times with NaCl (36 g.L^−1^) and dried for 30 min at 50 °C. Biofilms were stained for 15 min with 200 μL of Crystal Violet at 0.01%, rinsed three times with NaCl (36 g·L^−1^), and dried 10 min at room temperature. The quantification of biofilm was evaluated by releasing the stain from the biofilm with absolute ethanol for 10 min at 20 °C on an orbital shaker 120 rpm [34,35] and by measuring the absorbance of Crystal Violet solution at 595 nm.

### 2.10. Amoeba Against Bacterial Biofilm

A preformed biofilm of bacteria was established in NSS in 24 well plates (Corning Incorporated Costar^®^, New York, NY, USA), containing a sterilized glass coverslip into each well, to a final OD_600 nm_ of 0.3 for monospecies biofilms and 0.1 per strain for multispecies biofilms, similarly to our previous study, except that NSS was used instead of artificial sea water (ASW) [18]. After 24 h or 48 h of incubation at 20 °C in static conditions, 1 mL of NSS for the blank, 1 mL of amoeba-preconditioned supernatant, or 1 mL of amoeba suspension at 5 × 10^5^ cells/mL in NSS was added onto the preformed biofilms. The preconditioned supernatant was collected from a culture of amoeba at 5 × 10^5^ cell/mL in NSS, which was filtrated onto a 0.2 μm filter after 24 h of incubation. After 24 h of incubation at 20 °C in static conditions, cells were fixed using Formalin 3.7% for 3 h. The permeabilization and staining steps were performed as previously described [18]. Finally, the coverslips were mounted with a drop of ProLong™ Diamond Antifade before observation using CLSM. For biofilm of TC10 pX5-GFP, after the fixation step, the samples were directly mounted with the same Prolong^TM^ Diamond Antifade mounting medium. 

### 2.11. Evaluation of the Presence of Planktonic Bacteria

Preformed bacterial biofilms were performed in NSS in 24 well plates, containing a sterilized glass coverslip into each well, to a final OD_600 nm_ of 0.4 (0.1 per strain for multispecies biofilm). After 24 h of incubation at 20 °C in static conditions and after three washes, 1 mL of NSS for the blank, 1 mL of amoeba-preconditioned supernatant prepared as described above, or 1 mL of amoeba suspension at 5 × 10^5^ cell/mL in NSS was added onto preformed biofilms. After 24 h of incubation at 20 °C in static conditions, the supernatants of the biofilms were plated on VNSS agar before analyzing colony forming unit (CFU) enumeration. 

### 2.12. Data Extraction from Images and Statistics

At least three biological replicates and ten pictures per sample were performed for each experiment. The pictures were acquired under the 63x/1.40 Oil DIC objective by CLSM (Zeiss LSM 510, Göttingen, Germany). The biovolume of CLSM pictures was determined with the COMSTAT software developed in MATLAB R2015 a (Mathworks, Natick, MA, USA) [36]. To test for statistically significant differences (*p* < 0.05) between two conditions, a T-test was performed and a one-way analysis of variance including the Bonferroni post-test were performed between different time points, using SPSS 13.0 (IBM, Armonk, NY, USA).

## 3. Results

### 3.1. The Bacterial Strains Are Not All Digested and Follow Different Intracellular Paths within A. castellanii

In order to understand how marine bacteria could potentially survive in the presence of the predator *A. castellanii*, we first studied separately the interaction of four bacteria used in a previous study [18], *P. mediterranea* TC4, *Polaribacter* sp. TC5, and two different strains belonging to different species of *Shewanella,* TC10 and TC11 [14,15], with the amoeba, at a low bacteria-to-amoeba cells ratio. The objective was to analyze their interaction at the cellular level and more precisely to characterize the bacterial intracellular paths.

#### 3.1.1. *A. castellanii* interact with the Marine Bacteria TC4, TC5, TC10, and TC11

To study marine bacteria-amoeba interactions, we first tried to axenize amoebae (such as *Acanthamoeba griffini* CCAP 1501/4 and *Vahlkampfia dumnonica* CCAP 1588/9) isolated from marine environments recovered from the marine culture collection (Culture Collection of Algae and Protozoa CCAP/The Scottish Association for Marine Science (SAMS)), but without success. We then decided to perform our experiments with a well-known amoeba model *Acanthamoeba castellanii*, which has previously been isolated from marine environments and studied in interaction with marine bacteria such as *Vibrio* strains [1,10,11,25,37,38].

To perform the amoeba–bacteria interaction study, bacteria were grown beforehand planktonically, but a medium in which both partners could survive, without replicating, had to be selected. Among a set of media tested (data not shown), NSS, which tends to mimic the marine seawater composition, appeared to be the most promising one. NSS did not induce a significant decrease in the overall bacteria and amoebae number, and none of them grew either in this low nutrient solution (Supporting information Figure S2). The advantage of low nutrient media is that they tend to promote interactions between both partners [39]. 

To know if bacteria could exert a potential cytotoxic effect on the protozoan cells, propidium iodide was used on the protozoa incubated with each of the strains at MOI 100. The results showed that none of the bacteria triggered a cytotoxic effect on *A. castellanii*, similar to the non-infected cells and in sharp contrast with the formalin-killed control samples (Supporting information Figure S3).

With the purpose of evaluating the bacterial fate when cocultured with the amoeba, *A. castellanii* were cultivated in the presence of bacteria in NSS. At various time points bacteria cells were harvested from lysed amoeba cells for CFU enumeration purposes (Supporting information Figure S4). Our results showed that intracellular bacteria CFU decreased significantly for TC5, TC10, and TC11 by a factor 10 to 1000 depending on the bacteria (Supporting information Figure S4). For TC4, no bacteria could be detected, at first in contrast with the other strains as TC4 appeared sensitive to the triton treatment used for amoeba cell lysis (data not shown). To overcome this problem, which prevented the TC4 CFU enumeration when in interaction with the amoeba, we used a mechanical cell lysis approach. We were then able to detect TC4 CFU on plates, which confirmed that like for the others, the bacteria were alive within the amoeba. We observed a decrease of the TC4 concentration for the first 24 h of interaction. The CFU were thereafter stable until 48 h, suggesting a stable interaction between both partners.

Taken together, these results show that when cultivated alone in NSS, the overall proportion of amoeba and bacterial cells in the coculture experiments did not show variations over 48 h. However, when in interaction with the protozoa, no bacteria cytotoxic effect toward the amoeba was detected but there was an overall decrease of intracellular bacterial concentrations for TC4, TC5, TC10, and TC11, suggesting that some of the bacteria may have been phagocytized by the amoeba, and subsequently digested and/or released. 

#### 3.1.2. All the Marine Bacteria Are Phagocytized in Different Proportion and Persist Over 48 h within *A. castellanii*

**Figure 1:**
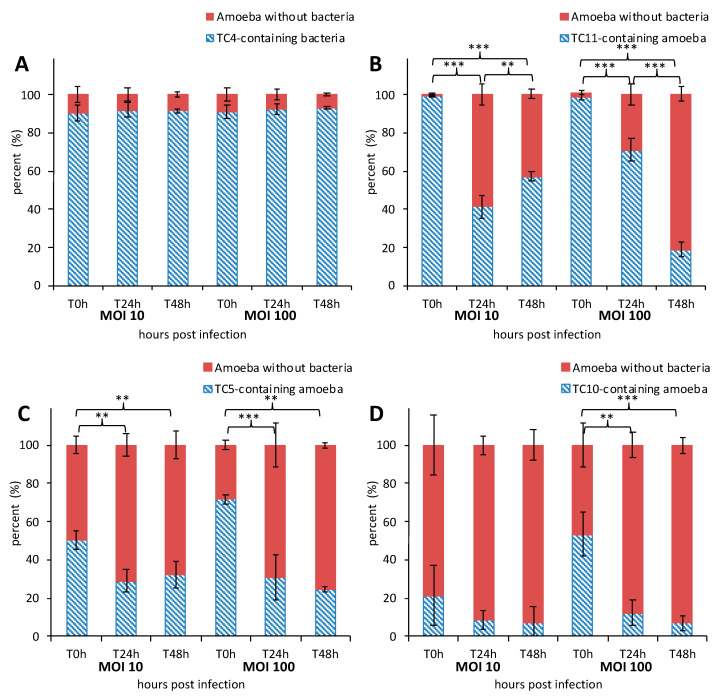
The marine bacteria were phagocytized and persisted within *Acanthamoeba castellanii* over 48 h. Kinetics of *A. castellanii* interaction with different marine bacteria was monitored over a course of 48 h at MOI (multiplicity of infection) 10 and MOI 100 by marine bacterial strains: *Persicivirga mediteranea* TC4 (**A**), *Shewanella* sp. TC11 (**B**), *Polaribacter* sp. TC5 (**C**) and *Shewanella* sp. TC10 (**D**). The percentage of amoeba containing bacteria was determined at T0 h, T24 h, and T48 h. Values are the means of three replicates, error bars represent standard deviations and asterisks indicate significant differences to the samples (** *p* < 0.01 and *** *p* < 0.001).

**Figure 2:**
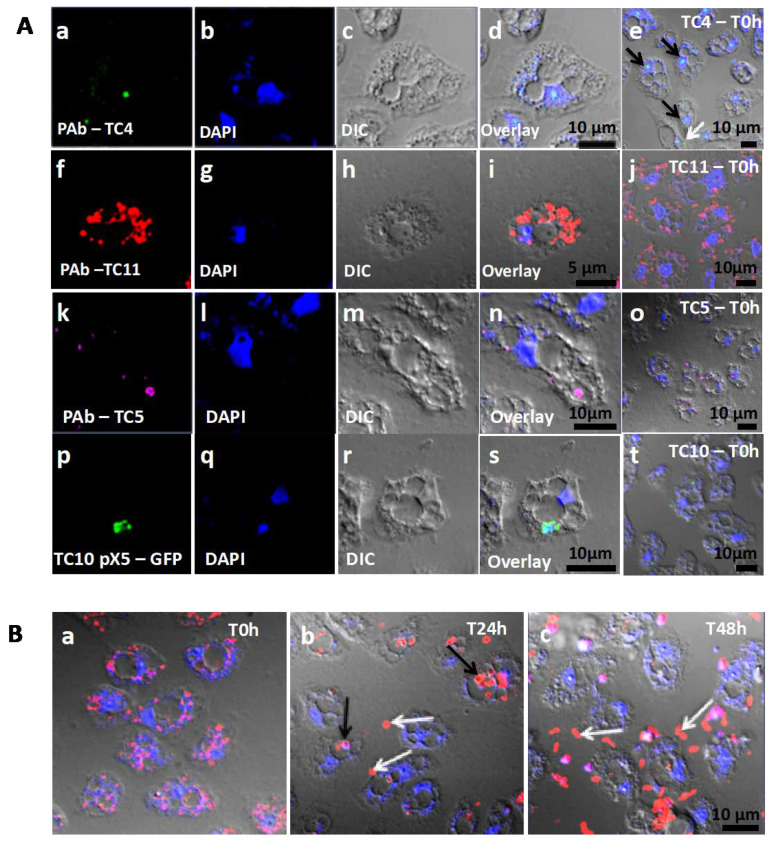
Bacteria were found in distinct locations within *A. castellanii*. (**A**) TC4 was located near the nucleus while TC5, TC10, and TC11 were cytoplasmic. *Persicivirga mediterranea* TC4 (**a**–**e**) was visualized using FITC (fluorescein isothiocyanate) (green)-conjugated polyclonal antibodies (PAb) (black arrows: nuclear bacteria; white arrows: cytoplasmic bacteria). *Shewanella* sp. TC11 (**f**–**j**) was labeled with Alexa Fluor 594 (red)-conjugated PAb. *Polaribacter* sp. TC5 (**k**–**o**) was observed with PAb conjugated to Alexa Fluor 633 (magenta). *Shewanella* sp. TC10 (**p**–**t**) harbored the plasmid pX5-GFP (green). For all bacteria, pictures were taken using CLSM (confocal laser scanning microcopy) at T0 h and at MOI (multiplicity of infection) 100; (**B**) TC11 was expelled in vesicles out of the cells over a course of 48 h. *Shewanella* sp. TC11 was observed in association with amoeba at MOI 100 at T0 h (**a**), T24 h (**b**), T48 h (**c**) (g and h black arrows: intracellular bacteria-filled vesicles; white arrows: expelled bacteria-filled vesicles). In each picture, amoebae were observed using DAPI (4′,6-diamidino-2-phénylindole) (blue) and with the corresponding DIC (differential interference contrast) picture of the confocal laser scanning microscopy (CLSM).

To characterize the mechanisms behind the phagocytosis events, the interaction between *A. castellanii* and the four bacterial marine strains were analyzed using CLSM. The monitoring of the co-incubated micro-organisms over the 48-h time period using CLSM shows that bacteria were phagocytized by *A. castellanii* in different proportions and appeared to display different behavioral patterns (Figure 1 and Figure 2). The evaluation of the presence of intracellular bacteria within *A. castellanii* indicated that most (i.e., 90%) of all amoeba contained *P. mediteranea* TC4 at both MOI 10 and MOI 100 (Figure 1A) at all time points. The fact that no increase or decrease of the number of infected cells was observed suggests some stability in the interaction over time. *Shewanella* sp. TC11 was present in most amoeba at T0 h (about 99%), but their numbers sharply decreased from T24 h at both MOI and reached about 57% and 19% at MOI 10 and 100, respectively at T48 h (Figure 1B). For *Polaribacter* sp. TC5, only 50% at MOI 10 and 72% at MOI 100 could be observed at most (Figure 1C). A significant decrease of infected cells was observed after 24 h at both MOI and this number stayed stable up to 48 h. The phagocytosis of *Shewanella* sp. TC10 was observed in about 21% or 53% of the cells at MOI 10 and 100 respectively from T0 h (Figure 1D). These numbers decreased afterward but like for the others, bacteria were still present within cells at T48 h. Therefore, these results show that *A. castellanii* phagocytosis occurred at different rate, and that marine bacteria may follow different intracellular paths, utilizing different mechanisms to circumvent rapid amoebal predation, since the four bacteria were still present at 48 h.

#### 3.1.3. TC4 Is Located within the Nucleolar Compartment

**Figure 3:**
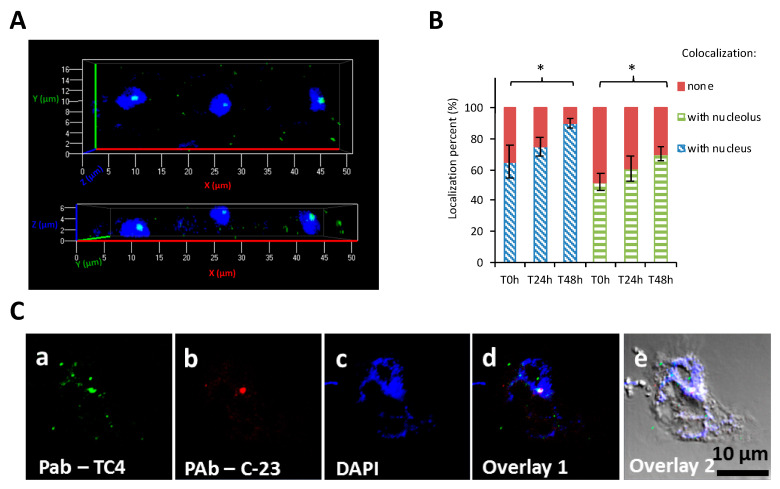
TC4 is located within the nucleolus. (**A**) *Persicivirga mediterranea* TC4 (**a**–**e**) was visualized using FITC (fluorescein isothiocyanate) (green)-conjugated polyclonal antibodies (PAb) and amoeba cells were labelled with DAPI (4′,6-diamidino-2-phénylindole). The 3D CLSM (confocal laser scanning microscopy) images of TC4 are displayed in 2 axial planes; (**B**,**C**) *Persicivirga mediterranea* TC4 (**a**–**e**) and amoeba were labelled as above. The nucleolin protein was labeled with C23 antibodies conjugated to CruzFluorTM594. The related evaluations in percentage of the colocalization with DAPI and C23 staining for each of the intracellular bacteria or group of bacteria at T0 h, T24 h, and T48 h. Values are the means of three replicates, error bars represent standard deviations, and asterisks indicate significant differences to the samples (* *p* < 0.05).

**Figure 4:**
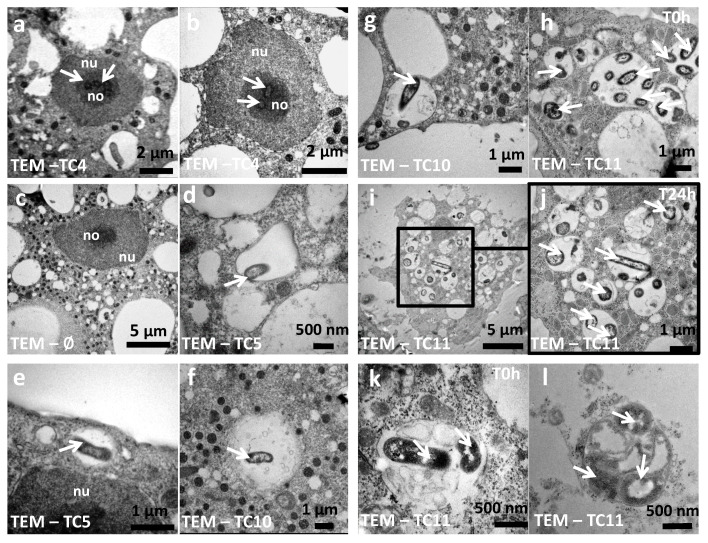
The bacteria and their phagosomes present different morphological characteristics within *A. castellanii*. TEM (transmission electron microscopy) photographs were taken at T0 h for *P. mediterranea* TC4 (**a**,**b**), *Polaribacter* sp. TC5 (**d**,**e**), *Shewanella* sp. TC10 (**f**,**g**), *Shewanella* sp. TC11 (**h**,**k**), at T24 h for *Shewanella* sp. TC11 (**i**,**j**) within amoeba or within a vesicle outside the amoeba cells (**l**) (white arrows: bacteria; nu: nucleus; no: nucleolus).

Analysis of CLSM images showed that most of the protozoan cells invaginated few TC4 (Figure 2Aa–e). Most of the TC4 immunostaining appeared to be associated with the DAPI staining (Figure 2Ae). The results showed that at T0 h more than 60% of the bacteria colocalized with DAPI and this number increased with incubation time, reaching 90% at 48 h (Figure 3A,B). It suggests that this bacterium is localized within or near the nucleus of the host cells. This observation could be made at all time points and at both MOI (data not shown), suggesting that the bacterium goes quickly (within the 3 h contact time) toward the nucleus area and stays in this intracellular niche for over 48 h. The 3D images displayed in two axial planes *y*/*x* and *z*/*x* show that TC4 staining was mostly detected in the middle of the DAPI staining area, suggesting a central location of the bacteria within the nucleus (Figure 3A). To confirm the location of TC4 in the amoeba nucleus, we performed TEM experiments. TEM photographs showed the presence of one to six electron dense structures of the size of a bacteria in the nucleus of the amoeba and more precisely in the electron-dense area of the nucleus, corresponding to the nucleolar compartment (Figure 4a,b). To verify this observation, antibodies directed toward the nucleolin protein known to be located within the nucleolus were used [33]. The results show that at T0 h, around 50% of bacteria colocalized with the nucleolin labeling, demonstrating that bacteria were already located within the nucleolus by that time (Figure 3B,C). At T24 h and T48 h, the proportion of bacteria colocalizing with the nucleolin labeling increased to reach over 60%. Therefore, more than half of TC4 are already associated with the host nucleolus within the first 3 h of contact time and stay there during at least 48 h. Thus, our data show that TC4 goes rapidly in the nucleolar compartment of the amoeba cells and stays there for a long period. 

An experiment where amoeba and TC4 were left in NSS or PYG for several weeks seems to indicate that after one month, TC4 were still observed within the nucleus of the amoeba (Supporting information Figure S4A,B). We also checked if TC4 could modify the amoeba behavior. We focused on amoeba DNA replication and encystment similar to previous studies [40,41]. These experiments seem to indicate that TC4, while in the nucleolar compartment, did not impact the amoeba DNA replication (Supporting information Figure S4C). TC4 did not induce or prevent amoeba encystment (Supporting information Figure S4D). Therefore, at this stage, TC4 has no known impact on the amoeba physiology/behavior and seems to establish a long-term relationship with its host.

#### 3.1.4. TC5, TC10, and TC11 are Localized in the Cell Cytoplasm and TC11 Appears to Be Expelled Outside the Protozoan Host at 24 h

Analysis of the CLSM images showed that TC11 were phagocytized in numerous phagosomes within the protozoan host at both MOI (Figure 2Af–j). At T24 h, structures resembling vesicles (also called pellets) containing bacteria appeared outside the amoeba (Figure 2Bb, white arrows and 4l) and at T48 h, bacteria were released from their vesicles (Figure 2Bc, white arrows). These results show that TC11 were expelled from the cells and this is further supported by the decrease of intracellular bacteria in the CFU experiments (Supporting information Figure S4). Similarly, analysis of the CLSM images showed that TC5 and TC10 were located in the cytoplasm (Figure 2Ak–t).

In addition, TEM images highlighted that TC11 was present in groups in numerous phagosomes, filling most area of the cell cytoplasm of amoeba (Figure 4h–k) whereas TC4, TC5, and TC10 appeared to have been invaginated in very small groups of bacteria in one or two phagosomes at the time (Figure 4a–g). Therefore, our results show that the four marine bacteria were phagocytized in different quantities/at different rates and they most probably follow different intracellular fate when they are phagocytized within *A. castellanii*.

#### 3.1.5. The Bacterial Strains Follow Different Intracellular Paths within *A. castellanii*

In order to better understand to which extent each of these marine bacteria could follow a different intracellular path, colocalization studies were performed using CLSM with an intracellular staining marker, the color becoming redder along with the vacuole acidification.

**Figure 5:**
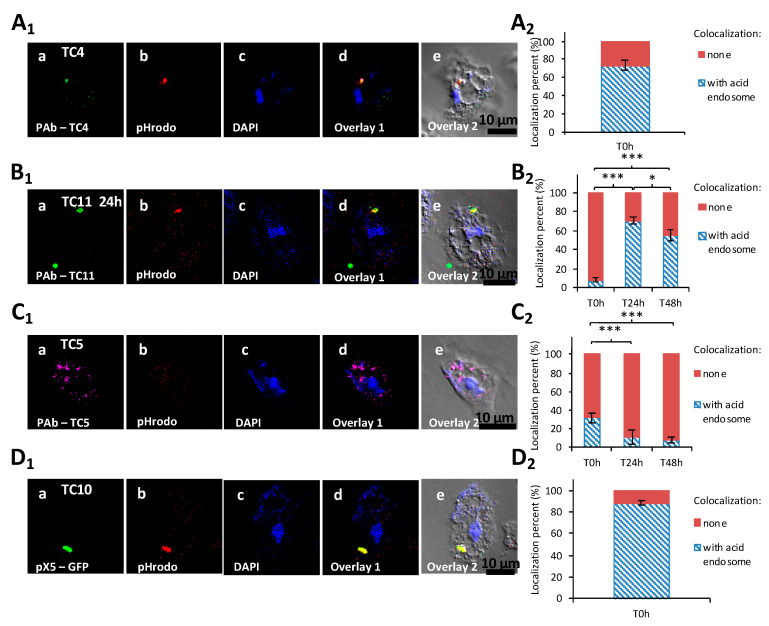
The four marine bacteria followed a specific intracellular trafficking within *A. castellanii*. Colocalization of *P. mediterranea* TC4 (**A_1_**), *Shewanella* sp.TC11 (**B_1_**), *Polaribacter* sp. TC5 (**C_1_**), and of *Shewanella* sp. TC10 (**D_1_**) was performed with acid endosome pHrodo staining at T0 h. Bacteria were labeled with either polyclonal antibodies conjugated with FITC (fluorescein isothiocyanate) (green) for TC4, Alexa Fluor 633 for TC5, Alexa fluor 594 (red) or CruzFluorTM 488 (green) for TC11, or with the pX5-GFP for TC10. DAPI (4′,6-diamidino-2-phénylindole) staining (blue) was used for the visualization of the host cell. Each set of pictures includes a superposition with the corresponding DIC (differential interference contrast) picture. (**A_2_**,**B_2_**,**C_2_**,**D_2_**) are the related evaluations in percentage of the colocalization with pHrodo staining for each of the intracellular bacteria filled-phagosomes at T0 h, or T0 h, T24 h, and T48 h. When the percentage of colocalization was high, suggesting a digestion for most bacteria in the cytoplasm, the time-points were not performed. Values are the means of three replicates, error bars represent standard deviations, and asterisks indicate significant differences to the samples (* *p* < 0.05 and *** *p* < 0.001).

In order to verify if the TC4 bacteria that remained in the cytoplasm were in acidified endosomes, cells were subjected to pHrodo™ Red Dextran staining. The results showed that about 70% of the cytoplasmic TC4 strains colocalized with pHrodo™ Red Dextran staining at T0, which highlights that lysosomal fusion with bacterial endosomes occurred for a majority of the cytoplasmic bacteria (Figure 5A_1_,A_2_).

The TC5, TC10, and TC11 bacteria were all in the cytoplasm. The staining of TC11-containing intracellular vacuoles with the pHrodo™ Red Dextran showed that compartments were slightly acidified at T0 h (8%) (Figure 5B_1_,B_2_). After 24 h and 48 h, about 70% and 60%, respectively, of the bacteria-containing vacuoles colocalized with the pHrodo™ Red Dextran staining. Therefore, acidification of the TC11 phagosomes occurs after 24 h or 48 h, which does not appear to prevent expulsion of bacteria-filled vesicles outside the host cells and subsequent release of the bacteria in the extracellular medium.

For *Polaribacter* sp. TC5 and *Shewanella* sp. TC10, while only about 30% of TC5 colocalized with pHrodo staining, more than 80% of TC10 did at T0 h (Figure 5C_1_,C_2,_D_1,_D_2_). These results show that TC10 is the bacteria the more rapidly digested within amoebae, while for TC5, the digestion is slower. This is also supported by CFU enumeration of intracellular bacteria (Supporting information Figure S4). Taken together, these results show that acidification occurs in different proportions and at different paces for each strain.

### 3.2. The Bacterial Biofilms Are Not Grazed Upon by A. castellanii

We have shown that the four bacterial strains are all phagocytized but are however able to resist to some extent to amoeba digestion. The objective here was to know if amoeba in the presence of sessile bacteria as monospecies or multispecies biofilms would allow either the formation of a stable multi-organism/multi-microbial biofilm, if the amoeba would be triggered to graze upon these bacteria or if another outcome would be unveiled. We therefore studied the interaction of preformed bacterial mono and multi-species biofilms in the presence of *A. castellanii* to analyze their interactions at the populational level. This would mean that the bacteria would be in a different physiological state but would also imply that the bacteria-to-amoeba ratio would be higher than in the previous experiments (MOI 10 or 100) nearing here 3000 bacteria for 1 amoeba.

#### 3.2.1. The Four Bacteria Are Able to form a Biofilm in NSS

To evaluate the abilities of marine bacterial strains to form a biofilm in NSS (a marine solution depleted of carbon sources), kinetics of biofilm formation were performed at 15 h, 24 h, 48 h, and 72 h using the Crystal Violet method. The results showed that while the strains were unable to grow planktonically in this medium, all the bacterial strains were able to form a biofilm in NSS with various efficiencies and paces (Supporting information Figure S6). The four bacterial strains formed a biofilm as soon as 15 h. A progression of the biofilm formation over the 72 h period was observed for all the strains, except for *Polaribacter* sp. TC5, whose biofilm remained stable for the 72 h period. The most important progression in biofilm formation was observed for *Shewanella* sp. TC10 and, to a lesser extent, *Shewanella* sp. TC11. Both strains presented significant difference in their biofilm formation between 15 h and 72 h as well as between 24 h and 72 h, which was not the case for TC4 and TC5. These results show that all the bacterial strains were able to form a biofilm in this low nutrient marine solution over a 72 h period.

#### 3.2.2. *A. castellanii* Induces Biofilm Detachment from the Surface

To evaluate the outcome of preformed monospecies bacterial biofilms in the presence or absence of *Acanthamoeba castellanii*, 24 h- and 48 h-old biofilms were selected for the grazing experiments as they had the highest OD (OD_595nm_ between 2.8 for TC4 at 24 h to 6.2 for TC10 at 48 h) with little sign of detachment. The preparation of the 24 h- and 48 h-old biofilms was followed by a wash and by the addition of, first, either the NSS medium or the amoeba, for another 24 h incubation.

**Figure 6:**
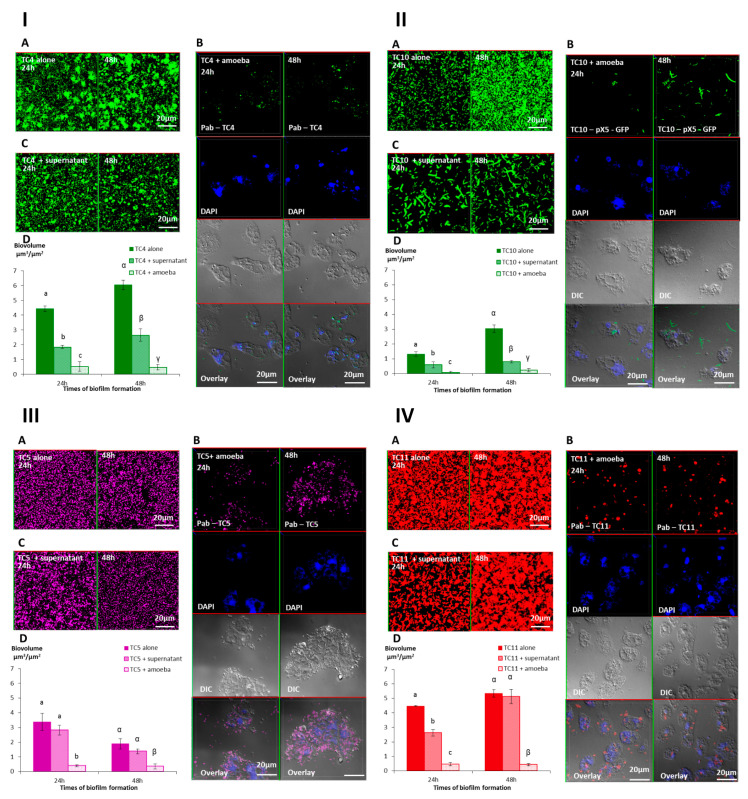
The presence of *A. castellanii* and its supernatant have an effect on *Persicivirga mediterranea* TC4 and *Shewanella* sp. TC10, *Polaribacter* sp. TC5, and *Shewanella* sp. TC11 biofilms. The 24 h- and 48 h-old *Persicivirga mediterranea* TC4 (**I**) and *Shewanella* sp. TC10 (**II**), *Polaribacter* sp. TC5 (**III**) and *Shewanella* sp. TC11 (**IV**) biofilms biovolumes were monitored using CLSM after the addition for an additional 24 h of NSS (nine-salt solution) (control) (**A**), amoebae (**B**), and the amoeba supernatant (**C**). Pictures are representative of three biological replicates. Bar plot represents the calculated biofilm biovolume in the different conditions relative to the incubation time (**D**). The biovolumes were evaluated by a COMSTAT algorithmic method. Values are the means of three biological replicates, error bars represent standard deviation, and significant differences between incubation times (*p*-value < 0.05) are displayed by different letters (**a**–**c**) in a same alphabet (a ≠ α).

The results showed that while in the absence of amoeba, all the biofilms presented numerous bacteria spread out on the surface, whereas most of the bacterial biofilms had disappeared in the presence of *A. castellanii* at both time points (Figure 6-IA,B,D,6-II-A,B,D,6-IIIA,B,D,6-IVA,B,D). When we looked closer at the remaining bacteria in the CLSM pictures, we noticed that *P. mediterranea* TC4 was mostly associated with the nucleus of *A. castellanii*. We also observed that extracellular *Shewanella* sp. TC10 were mostly under a filamentous form when *A. castellanii* was present, in sharp contrast with when TC10 were in the biofilm alone. When TC11 was in the presence of amoebae, we observed patches of bacteria in rounded shapes, which looked like bacteria-containing vesicles, around or within the host cells similar to what we previously observed in Figure 2. However, for TC5, numerous bacteria were associated with the few observed remaining amoebae, which looked bigger (about 60 μm versus 20 μm), as if they had been sticking to each other, and appeared flatter, showing necrotic signs, such as impaired membranes integrity (Figure 6-IIIB). This is in sharp contrast with the *A. castellanii* cells interacting with the other bacteria. Whether these observations are linked to a high phagocytosis rate of numerous bacteria by the amoeba, resulting in a toxic bacterial indigestion, or from a bacterial attachment onto dying amoeba bodies is not known. This result was quite surprising as at low bacteria-to-amoeba ratio, there was no cytotoxicity effect of TC5 on amoebae (Supporting information Figure S3). It is possible that with 24 h- or 48 h-old-preformed biofilms, the bacteria-to-amoeba ratio was unfavorable for the amoeba in the case of TC5 (relative MOI of 3000). Therefore, the interaction between TC5 biofilm and amoebae, whether it is through phagocytosis or extracellular contact-dependent cytotoxicity of a high number of TC5, triggered a cytotoxic effect on *A. castellanii*, resulting in a negative outcome for both partners. Overall, except for TC5, few bacteria were associated with the amoeba, which may suggest that bacteria may have detached from the surface rather than being phagocytosed and digested.

#### 3.2.3. The Supernatant of *A. castellanii* Induces Biofilm Detachment

In order to verify if bacterial phagocytosis and subsequent digestion were solely responsible for the bacterial disappearance or if bacterial detachment also occurred, we performed the same experiments with the addition of the amoebae supernatant onto the preformed bacterial biofilms. More precisely, we added on each of the 24 h- or 48 h-old biofilms, either the NSS medium as a control, the amoeba supernatant, or the amoeba suspension in NSS. The results showed that the biofilm biovolumes of TC4 and TC10 decreased significantly when the amoeba supernatant was added compared to when the bacteria were alone but were significantly higher than when the amoebae were present (Figure 6ID,IID). This suggests that the amoeba supernatant significantly affected the bacterial biofilm. For TC11, the same result was obtained, except that on a 24 h-old biofilm, the addition of the supernatant initially had a significant effect on the bacterial biovolume, which was then lost in the 48 h-old biofilm (Figure 6IVD). For TC5, no significant difference was found in the biofilm biovolumes with or without the amoeba supernatant (Figure 6IIID).

Interestingly, changes were observed in the biofilm structure and the physiological state of TC4 and TC10, in the presence of the amoeba supernatant compared to when only NSS was added, while no or little change was detected for the TC5 and TC11 biofilms. First, the TC4 patches observed in the biofilm reduced significantly in size when the supernatant was added (up to 15 μm versus up to 5 μm), which was not the case for the TC11-patches-containing biofilm (Figure 6IA,C). For TC10, longer filamentous bacteria could be observed after the addition of the amoeba supernatant versus of NSS (Figure 6IIA,C). Moreover, when amoebae were present on the TC10 biofilm, extracellular bacteria appeared filamentous (up to 10 μm) in contrast to the intracellular bacteria (2 μm) (Figure 6IIB). These physiological changes had also been previously observed when TC10 was grown as biofilms in the presence of TC11 or TC4 [18]. Overall, these results suggest that the supernatant may have an effect on TC4 and TC10 biofilm biovolumes and on the bacteria physiology.

**Figure 7:**
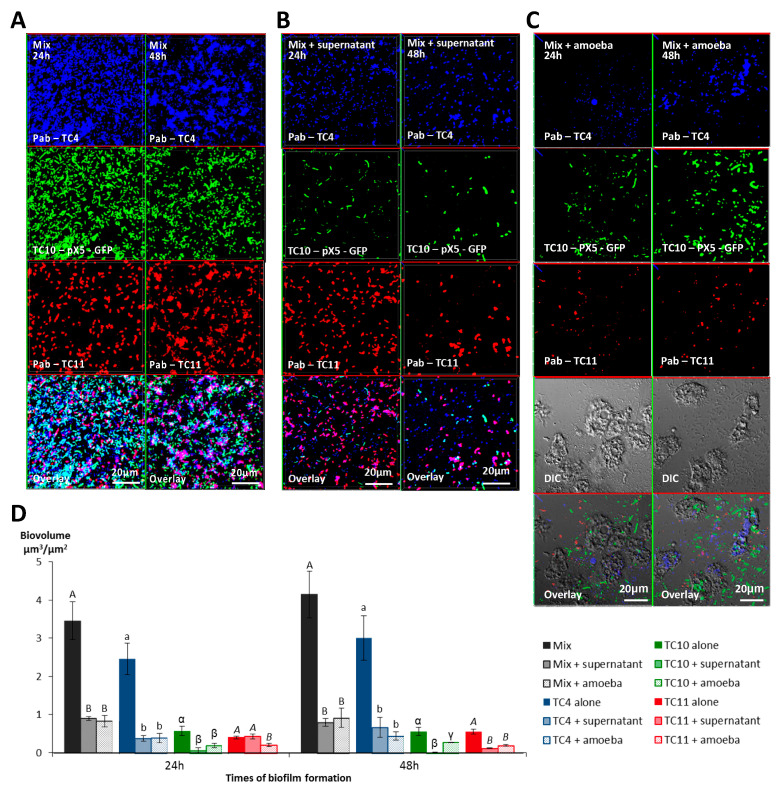
The presence of *A. castellanii* and its supernatant have an effect on *Persicivirga mediterranea* TC4, *Shewanella* sp. TC10, and *Shewanella* sp. TC11 in multi-species biofilms. Multispecies biofilm formation including *Persicivirga mediterranea* TC4, *Shewanella* sp. TC10, and *Shewanella* sp. TC11 in the presence of *A. castellanii* and *A. castellanii* supernatant. The formation of 24 h- and 48 h-old biofilms was monitored using CLSM (confocal laser scanning microscopy) after the addition for an additional 24 h of NSS (nine-salt solution) (control) (**A**), the amoebae supernatant (**B**), and the amoebae (**C**). Bar plots represent the calculated biofilm biovolumes in the different conditions (**D**). Pictures are representative of three biological replicates. Biovolumes were evaluated by a COMSTAT algorithmic method. Values are the means of three biological replicates, error bars represent standard deviation and significant differences between incubation times (*p*-value < 0.05) are displayed by different letters (**a**–**c**) in a same alphabet (A≠ a ≠ α≠ γ, B ≠ b ≠ β ≠ γ and A≠B, a≠ β).

We have previously shown that competition and/or cooperation controlled the interactions between these bacteria and that components can be secreted in multispecies biofilms while they were not in monospecies [18]. These interactions may be modified in the presence of external factors. The presence of amoebae could change the equilibrium of these bacterial interactions. In addition, multispecies biofilms have been shown to be more resistant to harsh conditions such as the presence of predators or toxic molecules [42]. In order to know if the presence of amoebae or its supernatant would have the same effect on each bacteria of the multispecies biofilm or if the bacterial interactions could modify this effect, we performed the same experiments with the mixed biofilm of *P. mediterranea* TC4, and *Shewanella* TC10 and TC11. *Polaribacter* sp. TC5 was not used in these experiments as its interaction with *A. castellanii* triggered a cytotoxicity effect on amoebae leading to necrotic features. Similarly, after 24 h and 48 h incubation, either NSS alone, *A. castellanii*, or the amoeba supernatant was added on the mono- and multispecies biofilms, which were incubated for an additional 24 h. Interestingly, the results showed that similar to our previous study, *P. mediterranea* TC4 grew better than the other strains in the three-species biofilm at both time points when they were cultivated in NSS without the amoebae or the amoeba supernatant (Figure 7A,D). Therefore, the NSS did not modify the predominance of TC4 (close to 3 μm3/μm2) in the three species biofilm that was observed in ASW in our previous study [18]. However, the TC10 biofilms seemed more inhibited in this mixed biofilm in NSS (below 1 μm3/μm2) than in ASW (above 1 μm3/μm2), while TC11 displayed, in both cases, a biofilm biovolume below 0.5 μm3/μm2. Nevertheless, when the amoeba supernatant was added, all the strains had a decreased biofilm biovolume, except for TC11 at 24 h only. At 48 h the amoeba supernatant had an impact on all the strains. The addition of the amoeba themselves did not induce a significant additional fall in the strains biovolumes except for TC11 at 24 h (Figure 7B–D). It seems that while the supernatant had no impact on the TC11 monospecies biofilm at 48 h, this effect was significant on TC11 in multispecies biofilm. This could suggest that the effect was somehow transferred to the TC11 biofilm in the presence of the other bacteria. Therefore, these results confirmed that amoeba and its supernatant induce bacterial biofilm detachment.

In order to confirm that the presence of the amoeba supernatant or the amoeba themselves on the monospecies or multispecies biofilms induced bacterial detachment, the non-adhered bacteria floating above the single or multispecies biofilms, which had all been treated similarly, were plated for CFU enumeration. First, we noticed that even when the bacteria were alone, after the addition of NSS, a substantial concentration of bacteria were found floating above biofilms (Supporting information Figure S7). In addition, this experiment showed that when the amoeba supernatant or the amoeba themselves were added, the dispersal was significantly more important for all the bacteria. The fact that no significant difference was found between the addition of the supernatant versus of the amoeba, which is less than one log difference, might be due to the sensitivity of the technique. Nevertheless, this experiment showed that a significant bacterial dispersal occurred when amoebae or amoeba supernatants were added to the single species and multispecies biofilms.

## 4. Discussion

**Figure 8:**
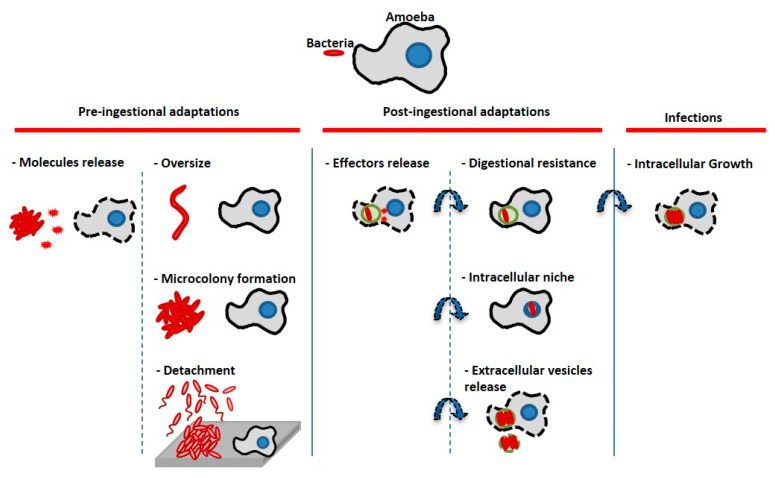
Bacterial escape mechanisms from amoeba predators/host cells (adapted from [5]). Bacteria can display pre-ingestional or post-ingestional strategies to escape the host cell digestion process. Bacteria can prevent ingestion or kill their host cells through molecules secretion, they can induce physiological modifications (changing size or regrouping) which prevent phagocytosis. Bacteria can also sense their predators and flee through biofilm detachment and/or swimming away (basically the inverse strategy of microcolony formation). Bacteria can be ingested by the amoeba, but they may secrete effector molecules to prevent phagolysosmal fusion and resist lysosomal digestion; they can find different intracellular niches to hide such as the nucleus or the nucleolus (TC4). They can also be expelled outside cells through vesicles secretion (*Shewanella* sp. TC11, *Legionella*, etc.). After having been able to escape the previous lysosomal digestion steps, some of them are also able to replicate for instance in the cytoplasm, the nucleus or in their host cell phagosomes. In our study, at least two of the bacteria seemed to display an escape mechanism: At the cellular level, TC4 was located in the amoeba nucleolus and TC11 was expulsed from the amoeba cells. At the population level, our hypothesis is that bacteria facing amoeba grazing may have developed large scale escape mechanisms in response to amoeba chemical cues, through detachment and/or swimming away.

The objective of this work was to study the interaction of amoeba with marine bacteria in order to understand how marine bacteria could survive in a microbial biofilm in the presence of a protozoan predator. If protozoa are bacterial grazers and part of the biofilm microbial communities at the same time, some bacteria must therefore display escape mechanisms from their predators. In this study, we first show that protozoan interactions with bacteria considered as non-pathogenic can unravel different intracellular paths, including intranucleolar localization and expulsion of bacteria-filled vesicles outside the protozoan cells, when we could have expected a quick bacterial digestion/grazing for all of them. Second, when amoebae are added to preformed monospecies or multispecies biofilms, the presence of amoeba or its supernatants induces biofilm detachment from the surface. Therefore, bacteria appear to display different escape mechanisms at the cellular level when in a low bacteria-to-amoeba ratio, but also at the population level, when they are grown as monospecies or multispecies biofilms. Figure 8 presents a summary of the bacterial escape mechanisms adapted and modified from the initial description from Matz et al. in 2005 [5], which includes the ones described in this paper. Despite an important diversity and abundance of heterotrophic protists such as protozoan amoeba present in the marine environment [13], these microorganisms and their interactions with bacteria are poorly studied and understood. They are most probably major actors in biofilms and the main predators of these ecosystems. They also could play an important role in selection and resistance of bacterial populations. Multispecies biofilms studies involving predatory eukaryotic cells are also very rare.

When the bacteria-amoeba interaction to a low cell-cell ratio was looked at, two of the bacterial species displayed escape mechanisms from the amoeba intracellular digestion. *P. mediterranea* TC4 was found within the nucleolar compartment of *A. castellanii.* To our knowledge, it represents a new location for an intracellular bacterium. It is the first description of an endonuclear and therefore endonucleolar bacteria among flavobacteria. In some cases, bacterial symbionts have been identified in the nucleus of protozoan cells (reviewed in [12]). However, due to the difficulty recovering non-culturable bacteria from this location, very few infection/interaction studies have been performed and reproduced, which usually lead to further molecular and/or cellular investigations [39,43,44,45,46]. For instance, the re-infection of an isolated protozoa by intranuclear symbiontic bacteria, which is able to recolonize again the nucleus of the cells of *Hartmannella* sp. and *A. castellanii* [39] or of some ciliates *Paramecium* [46,47], has been described. Intranuclear bacterial parasites have also been identified within mussels from deep-sea environments [48] or other invertebrates [49,50,51], and a few pathogenic bacteria such as *Burkholderia pseudomallei* [52] or obligate intracellular bacteria belonging to the *Rickettesia* genus [53,54,55] have been detected in the nucleoplasm of murine or human cells.

Studies on the mechanisms explaining how the bacteria reach the nucleus are very rare. In the case of *B. pseudomallei*, while bacteria are mostly cytoplasmic, a few dormant bacteria were found within the nucleus of human and murine epithelial cells, which they reach after escape and multiplication into the cytoplasm, and formation of actin comet tails to enter this location [52]. In the case of the symbiont *Candidatus Nucleicultrix amoebiphila* recovered from *Hartmannella* sp. isolated from an activated sludge sample, the purified bacterium was used for re-infection studies and was able to reach the nucleus of *Hartmannella* sp. within 4 to 6 h and quickly started replicating. They reached this location right after bacterial escape in the cytoplasm from endosomes fused with lysosomes. The bacteria became lytic for *Hartmannella* sp. FS5 and *A. castellanii* after 96 h, while being located within the nucleus. Infection was not observed for *Acanthamoeba* sp. UWC8 and *Naegleria gruberi* [39] suggesting it is a host cell-dependent mechanism. In our case, TC4 appears to find, in a similar time frame, a stable niche in the nucleolar compartment in which it stays for at least 1 month, without signs of replication and elimination or expulsion. Therefore, TC4 seems to establish a long-term relationship that would need further investigations to understand the mechanisms behind this intracellular path, and what this location brings to the bacteria [39]. No difference between MOI 10 and 100 was observed suggesting that the phagocytosis capacity of the amoeba cells was already at its maximum at MOI 10. Since a majority of the few cytoplasmic TC4 bacteria colocalized with acidified endosomes, it is possible that, either some bacteria are quickly digested during the infection process while others are routed to the nucleolus, or they manage to escape after lysosomal fusion into the cytoplasm before going to the nucleolus, as previously described [39]. The mechanism by which the TC4 bacterium goes from the endosomal to the nucleolus compartment remains to be discovered. Nucleolus is known to be often hijacked by effector proteins secreted by pathogenic intracellular bacteria such as *L. pneumophila*, as its first role is rRNA synthesis, ribosome biogenesis, mitosis regulation and cell proliferation, which bacteria may exploit for their own survival as do numerous viruses [56]. The intranuclear and even more intranucleolar location may represent the best protective niche for establishing symbiosis or parasitism within host cells, as bacteria are protected from cytoplasmic processes such as digestion [12]. Culturable bacteria such as TC4 may help in understanding how bacteria enter the nucleolus and stay there.

Another interesting intracellular fate found in this study is the one of *Shewanella* sp. TC11, which appeared to be found in expelled vesicles after 24 h infection of *A. castellanii*. The formation of vesicles or fecal pellets has been shown to represent an advantage against harsh conditions such as the presence of chlorine, antibiotics, and biocides, and allows bacterial survival [57,58,59]. It is also involved in spreading on long distances of respirable particles [57], or described as more infectious and as a vector to pass through the stomach and its acidity, before the establishment of an intestinal infection by *Vibrio cholerae* [9]. *A. castellannii* are able to produce both cysts, vesicles, and multilamellar bodies containing bacteria such as *L. pneumophila*, *Francisella tularensis*, or *Vibrio cholerae* [9,57,60,61,62,63,64,65]. Vesicles can be of different morphologies as described previously [10,60,66]. For instance, in the case of the protozoa *Tetrahymena pyriformis*, some vesicles contained amorphous material and fragments of membranes and others contained non-continuous membranous materials or whorls, while sometimes they lacked a defined membrane around them or were a mix of all above descriptions [57,60]. The ability of the bacteria to be packaged by the protozoa is known to be dependent on the bacterial strains, the protozoa, and the culture conditions [60,67,68,69]. In our case, the TEM picture of the Figure 4k,l shows that a membrane is surrounding the vesicle that contains at least one TC11 bacteria. This expulsion and propagation of bacteria-filled vesicles from protozoa could be a widespread phenomenon in environments including in the marine environment.

*Polaribater* sp. TC5 and most importantly *Shewanella* sp. TC10 were the least able to be phagocytized by *A. castellanii*. While few TC5 colocalized with acidified endosomes, a majority of TC10 did. One observation that could explain a poor phagocytosis is the fact that TC10 is able to filament which was not observed with the other bacteria. When either the filtered supernatants of *A. castellanii* or the amoebae themselves were inoculated with the bacteria, TC10 appeared as longer and bigger filaments. Within the cell, TC10 displayed smaller shape than filamentous bacteria found outside the cell. It is therefore possible that bacteria are able to shape filaments induced by protists by-products as a defense mechanism to avoid amoeba phagocytosis, which would up-take only the smaller bacteria as described previously [70]. Morphological plasticity as part of phenotypic heterogeneity has been poorly studied while it may play a functional role within isogenic bacterial populations (reviewed in [71]). Filamentous morphology is known to provide survival advantages in particular during environmental stress or the presence of antibiotics or predators (reviewed in [72]). While some intracellular replicating bacteria form filaments within their host cells, it has been shown that in order to survive in their environment, some opportunist bacteria can modify their morphologies when in the presence of their predator or predator by-products to avoid grazing [70,73,74]. The size and shape depend on the bacteria and preference for grazing on the protists [75,76]. The mechanisms behind it are still to be understood, as it is not clear what really triggers the filamentation, unavailability of some nutrients, or molecules produced by the protists, such as chemical molecules, debris, or metabolic wastes, or both. At last, *Polaribacter* sp. TC5 appeared to resist or to slow down the rate of phagosomes acidification as very few bacteria at 24 h and 48 h colocalized with pHrodo compared to TC10 at T0 h. Although the decrease in intracellular TC5 CFU appeared the slowest, it is surprising that the decrease in the proportion of infected amoeba was similar to TC10. The bacteria may not have withstood acidification of their compartments similarly. It is well known that some bacteria can resist acidified environments and that acidification of phagolysosomes is not the only cause of bacterial death [77]. Overall, if we consider that part or most of the bacteria are being digested, which is supported by the decrease of the number of infected amoeba over time, as well as the decrease of the intracellular bacteria CFU, the digestion process, occurring through most likely different mechanisms, still allows the observation of bacteria at 48 h.

When we studied the interaction between monospecies or multispecies bacterial biofilm and the amoeba, with the objective to assess the impact of grazing by the free living amoeba *Acanthamoeba castellanii* on single and multi-species biofilms, we first showed that all biofilms were greatly eliminated from the surface during the 24 h interaction with the amoeba, most probably through detachment. We also showed that amoeba supernatant triggered the detachment of bacterial strains from the surface, suggesting that *A. castellanii* may produce chemical cues that may induce bacterial dispersal from monospecies and multispecies biofilms. Many organisms are known to produce antifouling compounds that inhibit or induce the dispersion of the biofilm [78,79,80]. Therefore, it is possible that another escape mechanism was observed in response to amoeba chemical cues. This would need to be further investigated. The fact that filamentous structures appeared for *Shewanella* sp. TC10 in the presence of amoebae and of amoebae supernatants is another indication that bacterial sensing of the amoeba presence may trigger modifications of bacterial morphology and behavior. *Acanthamoeba* sp. are known to be very efficient predators in feeding on sessile bacteria, as it does not belong to swimmers [81,82]. Therefore, it is possible that bacteria were able to sense the presence of their predator, either directly or indirectly, through chemical cues present in the supernatant, which induced dispersion of the biofilms. In the three-species biofilm, the amoeba supernatant was able to induce the detachment of all bacteria including the one, TC11, which was less sensitive to the amoeba supernatant in their monospecies biofilm. This may suggest that some kind of chemical communication took place to induce its detachment. The amoeba spotting by bacteria have been demonstrated with various strains of amoeba and bacteria. For example, when *Pseudomonas fluorescens* is exposed to the *A. castellanii* supernatant, the production of toxins pyrrolnitrin, 2,4-diacetylphloroglunicol (DAPG), and hydrogen cyanide, is increased [4,83,84]. This mechanism has been shown to be used by bacteria in response to a predator to avoid phagocytosis or development of filamentous morphotypes [5,70,72,85,86,87,88], which can be growth rate or quorum sensing dependent [89,90,91]. These chemical signals exchanged between organisms have been named “Kairomones” as they can benefit the recipient organism while not being beneficial for the producer [85,92]. For instance, the amoeba *Amoeba proteus*, which feeds on ciliates, is known to produce kairomones that induce a size increase of the ciliate *Euplotes octocarinatus* and an avoidance behavior with *E. octocarinatus* and *E. daidales* [93].

At last, *Polaribacter* sp. TC5 appears to possess internal fighting capabilities. First, it has been shown that in a four species biofilm, this strain outcompetes all the other bacteria [18] and can secrete molecules in its supernatant that can inhibit TC10 and TC11 growth in biofilm. Here, we also show that this bacterium is able to trigger a very important contact dependent cytotoxicity effect on amoeba at the populational level. At low bacteria-cell ratio, this bacterium seems to have the ability to limit acidification of its endosomes in a more important manner than its counterparts. It has recently been described that members of the *Flavoacteriaceae* such as the *Polaribacter* may have the capacity to secrete very diverse metabolites, in particular antimicrobial, antioxidant, and cytotoxic compounds, and molecules capable of degrading a number of complex polysaccharides and other exopolymers [94,95,96]. However, very few functional studies have been performed on this group of bacteria.

## 5. Conclusions

The overall objective of this study was to try answering the following conundrum: If protozoa are bacterial grazers and part of the biofilm microbial communities, how do bacteria survive in the presence of their predators in biofilms? We first looked at the interactions at a low bacteria-to-amoeba ratio. We showed that, first, all the bacteria are phagocytized within the protozoan cells and that bacterial uptake occurs at very different efficiencies; second, none of them are totally eliminated by *A. castellanii* during the 48 h incubation; and third, each bacteria follows a singular intracellular fate. Among these intracellular paths, *Shewanella* sp. TC11 appears to be expelled from the amoeba in vesicles at 24 h. We also made the original observation of an intranucleolar bacteria (*P. mediterranea* TC4) within a eukaryotic host cell. We then studied the bacteria–amoeba interaction at higher cell-cell ratio, between preformed monospecies and multispecies biofilms and *A. castellanii.* We showed that, first, all biofilms detached from the surface during the 24 h interaction in the presence of amoeba or its supernatant. We also showed that *Polaribacter* sp. TC5, which have been shown to display inhibitory mechanisms toward other bacteria in multispecies biofilms [18], triggered a cytotoxicity effect on the amoeba when the bacteria had grown as a biofilm and thus presenting a higher bacteria-to-cell ratio. This represents another way to avoid bacterial grazing by the protozoa. Therefore, our findings show that protozoan interactions with bacteria considered as non-pathogenic can unravel different intracellular paths and escape mechanisms when we could have expected a quick bacterial grazing for all of them. This study highlights the diversity of interactions between eukaryotic cells and bacteria that could exist in the marine environment. It also allows to comprehend how bacteria survive in the presence of their predator through various escape mechanisms at the cellular or at the population level. A better understanding of the regulation of cell detachment and the identification of the signal involved in this detachment, would give crucial information for potential strategies to control mono- but also multi-species biofilms, in a variety of sectors of activity.

## Supplementary Materials

The following are available online at https://www.mdpi.com/2076-2607/8/12/1982/s1, Figure S1: Specificity of the antibodies used in this study against Acanthamoeba castellanii, Figure S2: The NSS medium does not trigger major variations of cell numbers over 96 h, Figure S3: None of the 4 marine strains are cytotoxic towards A. castellanii, Figure S4: The TC4, TC5, TC10 and TC11 intracellular concentrations decrease during A. castellani interaction, Figure S5: Persicivirga mediterranea TC4 is found within Acanthamoeba castellanii for at least a month, Figure S6: All the bacteria are able to grow as biofilms in NSS, Figure S7: The amoeba and its supernatant induce important detachment of living bacteria from mono and multispecies biofilms.
