# Supplementary material for: Marine Bacteria Display Different Escape Mechanisms When Facing Their Protozoan Predators
Source: Microorganisms. 2020 Dec 12;8(12):1982. doi: 10.3390/microorganisms8121982 (PMC7763514; doi:10.3390/microorganisms8121982)
Supplement: Supplementary file 1 [file microorganisms-08-01982-s001.pdf]

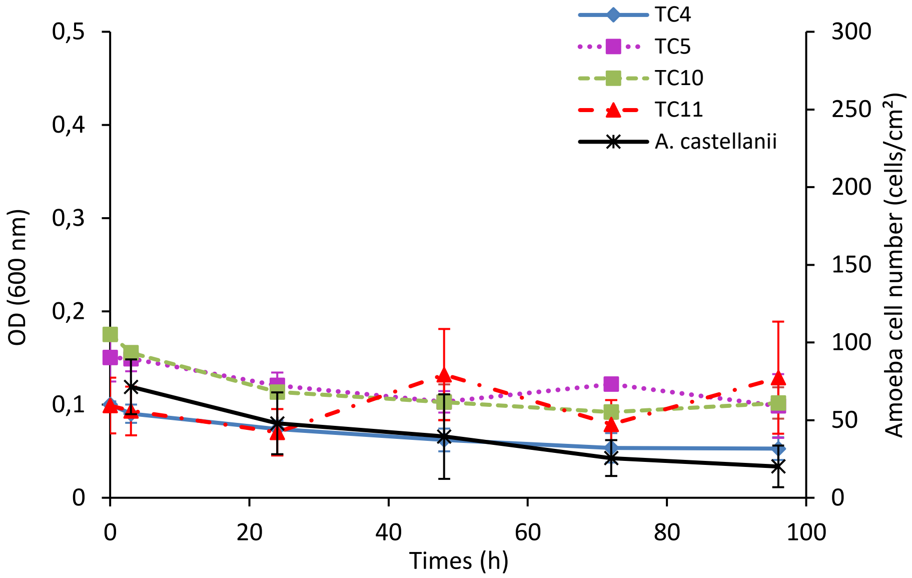

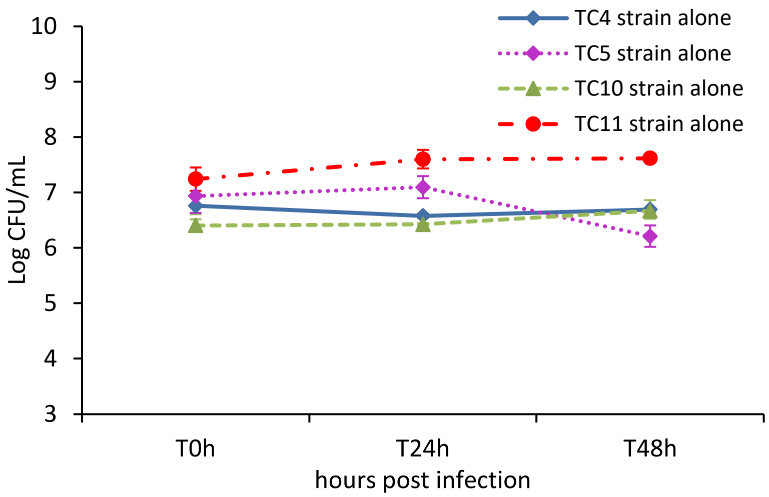

Supporting information Fig. S1 : The NSS medium does not trigger major variations of cell numbers over 96h. Growth kinetics in NSS of *P. mediterranea* TC4 (blue line), *Polaribacter* sp. TC5 (purple dot line), *Schewanella* sp. TC10 (green dash line), *Schewanella* sp. TC11 (red dash and dot line) and *A. castellanii* (black line) were evaluated using an inverted optical microscope for the amoeba and by OD_{600nm} measurement over a course of 96h (A) and by CFU over a course of 48h (B). Error bars show standard deviation based on three independent experiments.

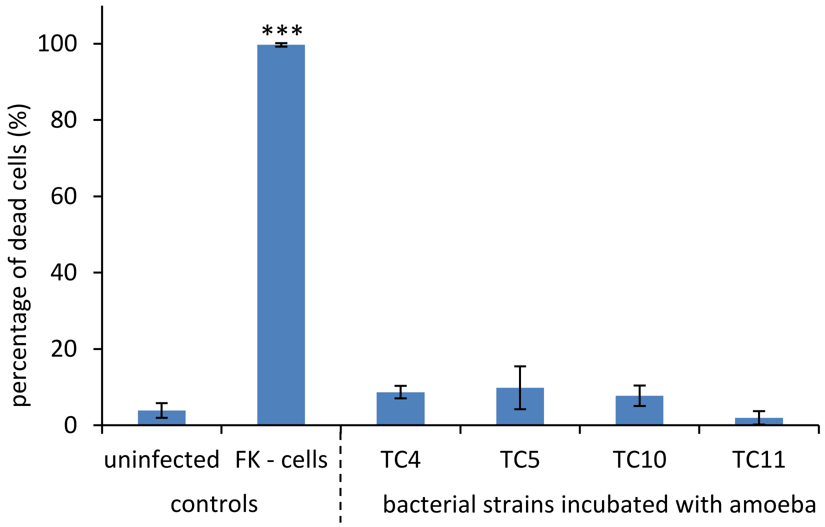

Supporting information Fig. S2 : None of the 4 marine strains are cytotoxic towards *A. castellanii*. *Persicivirga mediterranea* TC4, *Polaribacter* sp. TC5, *Schewanella* sp. TC10 and *Schewanella* sp. TC11 were inoculated in presence of *A. castellanii* and a cytotoxicity assay was performed 24h post infection using Propidium Iodide (PI). The number of dead amoeba cells were evaluated for *A. castellanii* in incubated with bacteria. The 2 controls includes non-infected amoeba and Formalin-killed(FK) amoeba. Values are the means of three replicates and error bars represent standard deviations. The results are representative of three different experiments.

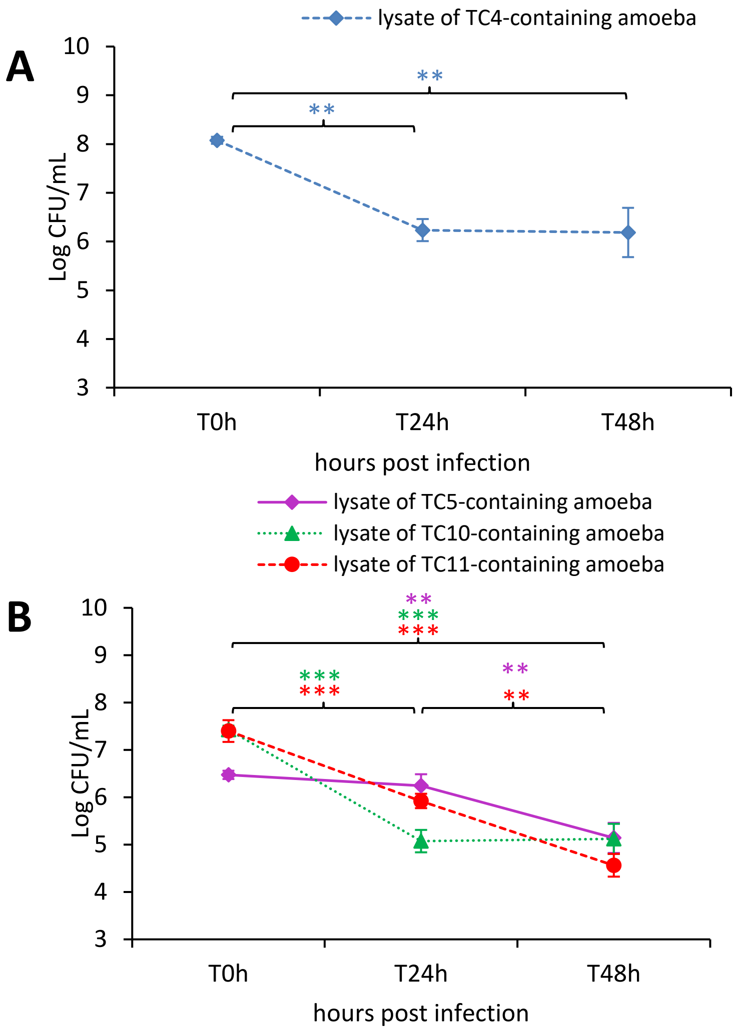

Supporting information Fig. S3 : The TC4, TC5, TC10 and TC11 intracellular concentrations decrease during *A. castellani* interaction. *Percisivirga mediterranea* TC4 (blue-full line), *Polaribacter* sp. TC5 (pink-full line), *Schewanella* sp. TC10 (green-dotted line) and *Schewanella* sp. TC11(red-dashed line) were cocultured with *A. castellanii* at MOI 100 and the amoeba lysates in presence of each bacteria were analyzed by CFU numeration at T0h, T24h and T48h by mecanical lysis (A) or chemical lysis (B). Values are the means of triplicate samples, and error bars represent standard deviations. The results are representative of three different experiments.

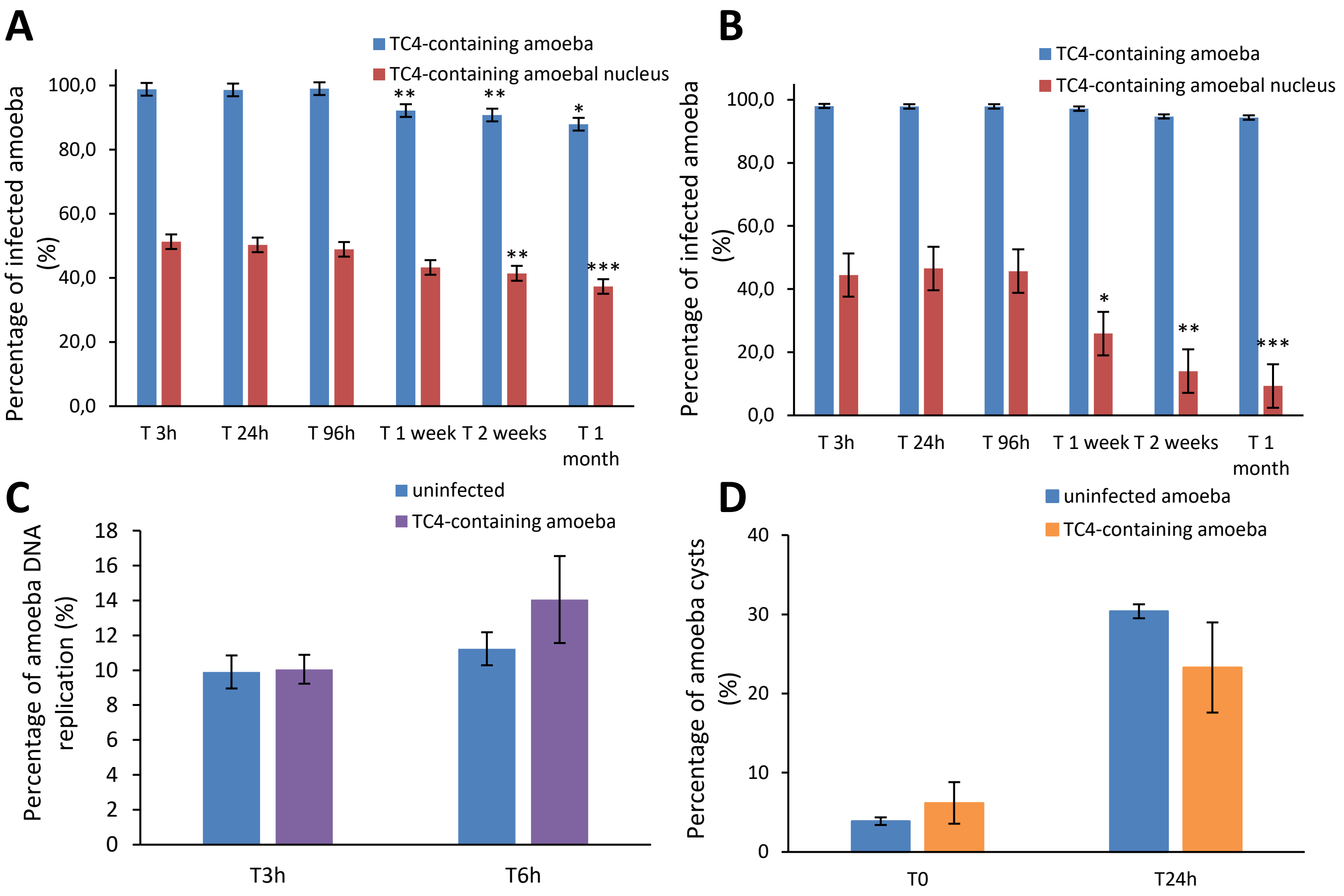

Supporting information Fig. S4. *Persicivirga mediterranea* TC4 is found within *Acanthamoeba castellanii* for at least a month. Kinetics of *A. castellanii* infection by TC4 was monitored over a course of 1 month at MOI 100 in NSS (A) and PYG (B). Percentage of amoeba replication during the infection by TC4 in PYG (in order to allow cell multiplication and therefore DNA replication) (C). Percentage of amoeba cysts after infection by TC4 in NSS at 24h and 48h (D). Values are the means of three replicates, error bars represent standard deviations and asterisks indicate significant differences between the same condition a T3h (P: *<0.05, **<0.01 and ***<0.001).

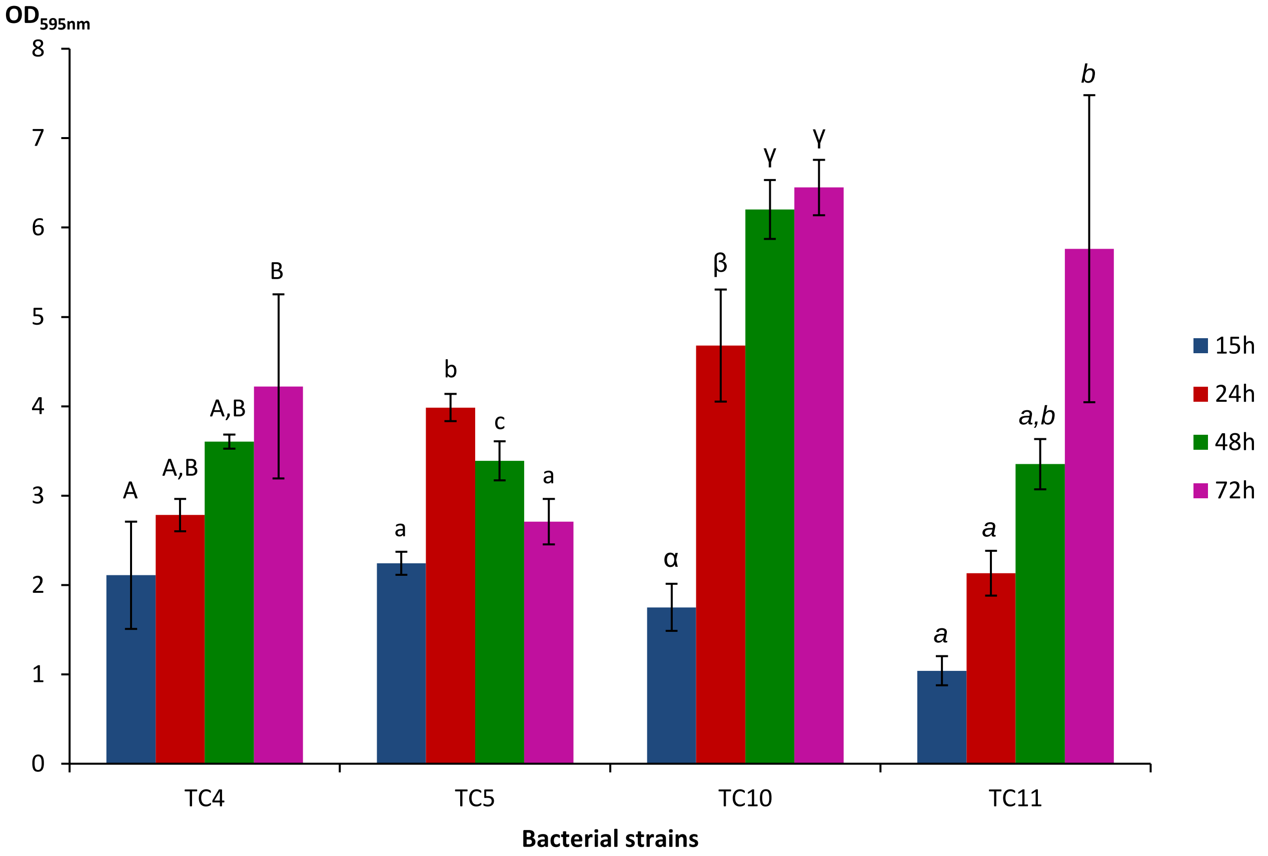

Supporting information Fig. S5. All the bacteria are able to grow as biofilms in NSS**.** Kinetic of the biofilm formation of *Percisivirga mediteranea* TC4, *Polaribacter* sp. TC5, *Shewanella* sp. TC10 and *Shewanella* sp. TC11 on microplates measured with Crystal Violet after different incubation times (15h, 24h, 48h and 72h) in NSS. Values are the means of three technical replicates representative of three biological replicates, errors bars represent standard deviation and significant differences between incubation times (p-value<0.05) are displayed by different letters (A versus B) in a same alphabet.

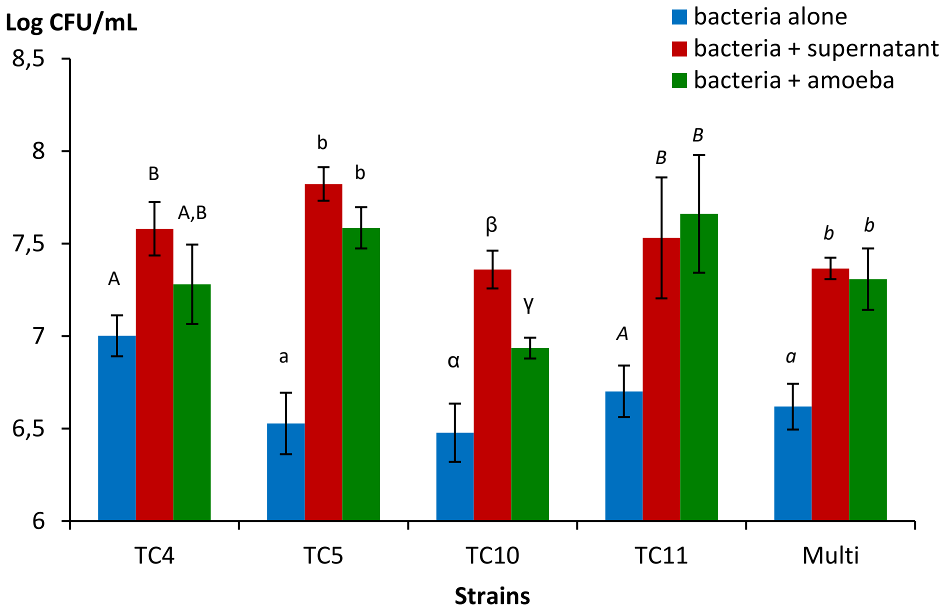

Supporting information Fig. S6**.** The amoeba and its supernatant induce important detachment of living bacteria from mono and multispecies biofilms. Concentrations of extracellular planktonic bacteria collected from the biofilms of *Persicivirga mediterranea* TC4, *Polaribacter* sp. TC5, *Shewanella* sp. TC10, *Shewanella* sp. TC11 and of the TC4, TC10 and TC11 multispecies biofilms in presence of the amoeba and the amoeba supernatant have been determined through CFU numeration. After 24h of biofilm formation, samples were washed and either NSS (control), the amoeba or the amoeba supernatant was added and incubated for an additional 24h. Serial dilution and plating of the different biofilm supernatants were performed on VNSS. Values are the means of three technical replicates representative of three biological replicates, error bars represent standard deviation and significant differences between incubation times (p-value<0.05) are displayed by different letters (a,b,c) in a same alphabet (A≠a≠α≠*A*≠*a*).

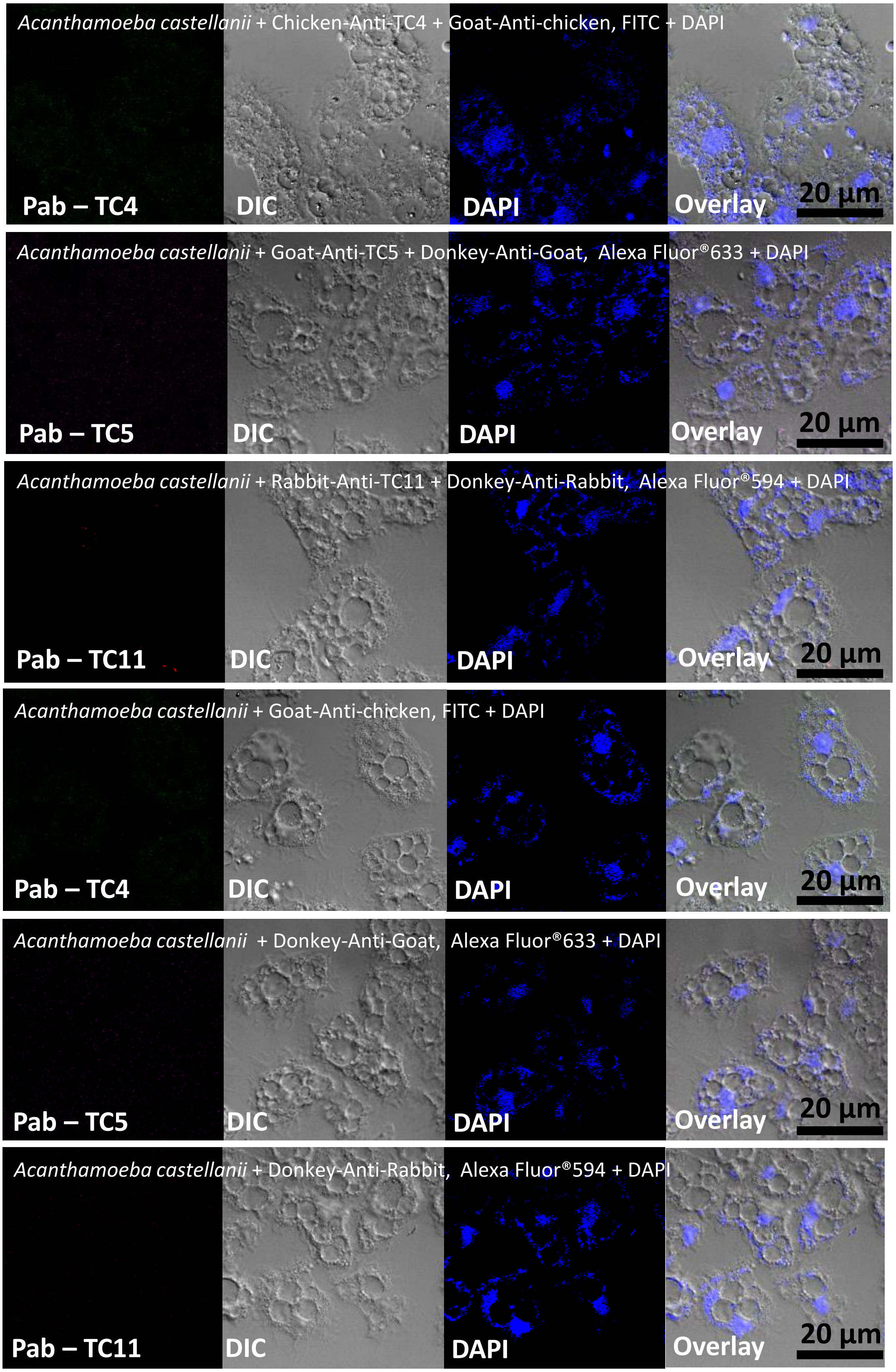

Supporting information Fig. S7 : Specificity of the antibodies used in this study against *Acanthamoeba castellanii.*
